# Regular *Plasmodium falciparum* importation onto Bioko Island, Equatorial Guinea, hampers malaria elimination from the island

**DOI:** 10.1371/journal.pgph.0004999

**Published:** 2025-08-19

**Authors:** Thomas C. Stabler, Ankit Dwivedi, Bing Guo, Biraj Shrestha, Sudhaunshu Joshi, Matilde Riloha Rivas, Olivier Tresor Donfack, Carlos A. Guerra, Guillermo A. García, Claudia Daubenberger, Joana C. Silva

**Affiliations:** 1 Department of Medical Parasitology and Infection Biology, Swiss Tropical and Public Health Institute, Basel, Switzerland; 2 University of Basel, Basel, Switzerland; 3 Institute for Genome Sciences, University of Maryland School of Medicine, Baltimore, Maryland, United States of America; 4 Department of Microbiology and Immunology, University of Maryland School of Medicine, Baltimore, Maryland, United States of America; 5 Malaria Research Program, Center for Vaccine Development and Global Health, University of Maryland Baltimore, Baltimore, Maryland, United States of America; 6 Equatorial Guinea Ministry of Health and Social Welfare, Malabo, Equatorial Guinea; 7 MCD Global Health, Malabo, Equatorial Guinea; 8 MCD Global Health, Silver Spring, Maryland, United States of America; 9 Global Health and Tropical Medicine (GHTM), Instituto de Higiene e Medicina Tropical (IHMT), Universidade NOVA de Lisboa (NOVA), Lisbon, Portugal; London School of Hygiene & Tropical Medicine, UNITED KINGDOM OF GREAT BRITAIN AND NORTHERN IRELAND

## Abstract

The Bioko Island Malaria Elimination Project (BIMEP) has made significant progress in reducing the prevalence of *Plasmodium falciparum* on Bioko Island, Equatorial Guinea. However, like other malaria endemic islands like São Tomé and Principe and Zanzibar, Tanzania, elimination efforts are hampered by imported infections. In an effort to understand the local transmission dynamics and the influence of importation on Bioko Island’s *P. falciparum* population, whole-genome sequences were generated from field samples collected during the BIMEP’s 2019 Malaria Indicator Survey (MIS). Within the sub-Saharan African context, we observed Bioko Island parasites did not significantly differentiate from nearby continental neighbors. Among Bioko infections, within-host diversity and the quantity of polyclonal infections appear similar to an area of moderate malaria transmission. However, we observed higher than expected genetic diversity among Bioko parasites, similar to high transmission areas, suggesting imported strains are contributing to transmission on the island. Among Bioko’s closest geographical neighbors, the flow of parasites with Bioko appeared more pronounced with the Gabonese parasite population, implying more importation may be coming from this region than others. Overall, despite significant investment in malaria control, results illustrate the challenges of eliminating malaria without both interrupting local transmission and accounting for importation from higher transmission areas, likely due to human migration. For there to be sustained progress towards elimination, the BIMEP needs, if feasible, to conduct targeted interventions of outgoing/incoming travelers, expand malaria control interventions to the continental region of Equatorial Guinea, and ideally conduct cross-border interventions in collaboration with the malaria control program in Gabon.

## Introduction

In the effort to eliminate malaria, islands hold a distinct advantage over malaria-endemic areas with land borders: their geographic isolation should, in theory, provide a barrier to parasite importation. With the introduction of effective control/prevention methods, this should facilitate rapid progress to malaria elimination. This has been observed in Sri Lanka and Cabo Verde, where no autochthonous cases have been reported since 2009, in the former, and 2017, in the latter, and which were declared malaria free in 2016 and 2024, respectively [1–3]. Unfortunately, for other island contexts, like Zanzibar and São Tomé and Principe, malaria continues to persist despite significant decreases in malaria prevalence [4–7]. Parasite migration, through host and/or vector movement, poses a significant obstacle to malaria elimination [8,9]. Critically, the identification of factors preventing malaria elimination on island settings could provide valuable evidence for malaria control programs to distribute their resources.

**Fig 1:**
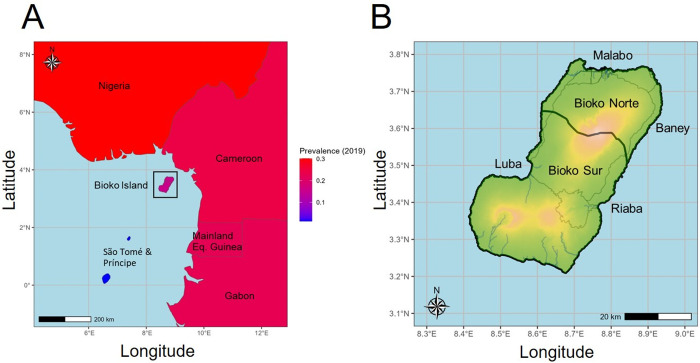
Map of Bioko Island, Equatorial Guinea. **A.** Geographical location of Bioko Island within the Gulf of Guinea. Color gradient denotes the estimated country-wide P. falciparum prevalence in 2019 reported in the 2023 World Malaria Report. Bioko Island color was amended to show island-wide prevalence as measured from the 2019 MIS. **B.** Detail of Bioko Island with province borders (black lines), roads (grey lines), rivers (blue lines) and elevation (green/red gradient – red denotes higher elevation). Shapefiles were obtained from the GEOs library (https://libgeos.org/).

Bioko Island, Equatorial Guinea, is located 32 km west of the coast of Cameroon and has historically high levels of malaria transmission [10] (Fig 1A). Intensive malaria control interventions have been conducted on the island since 2004 by the Bioko Island Malaria Elimination Project (BIMEP) (formerly the Bioko Island Malaria Control Project – BIMCP), which reduced malaria prevalence from 43.3% in the early 2000s to 10.5% by 2016 [10]. Despite continued interventions, which also resulted in significant reductions in malaria mortality on the island [10,11], malaria prevalence has fluctuated around 10–12% since 2016 [11,12]. Epidemiological, questionnaire-based studies by BIMEP demonstrate strong associations between on-island infections and recent travel to mainland Equatorial Guinea, suggestive of case importation, and identifying host movement as a potential source of parasite immigration to the island [11,13–15]. Further, as part of the COVID-19 pandemic response, a travel moratorium was implemented country-wide in Equatorial Guinea and without which it is estimated malaria infections would be 9% higher in communities with more reported travel [16]. However, the contribution of parasite migration to the composition of the Bioko Island parasite population remains to be validated and characterized molecularly.

The mainland region of the country, Rio Muni, has higher malaria prevalence than Bioko Island and has not had significant malaria control campaigns since 2011 due to funding constraints [17]. Cameroon and Gabon, neighbors flanking Rio Muni, are hyper-endemic regions with year-round transmission and high parasite genetic diversity [1,18,19]. If migration from the continent to Bioko Island is high, the island’s *Plasmodium falciparum* population, the predominant parasite species causing malaria [1], is expected to have a similarly high genetic diversity and little differentiation from Cameroon and Gabon. However, if the ocean forms a significant geographical barrier, and importation does not significantly contribute to Bioko’s malaria transmission and genetic diversity, then its *P. falciparum* population might differ significantly from those on the mainland.

Bioko Island is composed of two provinces (Bioko Norte and Bioko Sur) and four districts (Malabo, Baney, Luba and Riaba) (Fig 1B); Malabo City, the capital of Equatorial Guinea, located in Malabo district, Bioko Norte, harbors 90% of the island’s population of 270,000 people [11,20,21]. Historically, the greatest reductions in malaria burden have been achieved in rural communities, especially in the Baney and Luba districts [10]. However, rural residents also account for most of the on-island travel, Malabo being the primary destination for employment and education [14]. Considering the disparate human distribution and reported travel, more than one parasite subpopulation may exist. Conversely, importation and inter-island connectedness may be sufficiently high as to prevent differentiation and the formation of subpopulations.

The use of whole genome sequence data to estimate population genetic diversity and relatedness among *P. falciparum* strains can provide a highly nuanced assessment of the impact of control interventions over time and illuminate parasite transmission routes [22,23]. Consequently, WHO encourages malaria control programs to incorporate parasite genome sequencing technologies among their molecular surveillance techniques [24]. Previous studies have investigated demography of *P. falciparum* populations using whole genome data in sub-Saharan African [25]; however, the vastness of this region has resulted in geographic “pockets” with incomplete characterization of circulating genetic diversity. Especially in areas with active malaria control campaigns but limited history of molecular surveillance, characterization of *P. falciparum* genome-wide genetic variation and estimating genetic relationships among isolates can provide insights into the response of a parasite population to ongoing interventions and provide valuable data to inform future approaches to parasite control [22,26–28].

Here, we aim to characterize the genetic diversity and population structure of *P. falciparum* on Bioko Island and determine its relationship with parasite populations in neighboring regions on the mainland. Dried blood spot (DBS) samples obtained from a subset of participants in the BIMEP’s 2019 malaria indicator survey (MIS) were used to generate whole-genome sequence (WGS) data. Differentiation between Bioko Island and continental *P. falciparum* parasite populations was estimated, local transmission dynamics studied, by describing island-specific markers and relatedness between *P. falciparum* isolates.

## Methods

### Ethics statement

Ethics approval for the annual implementation of the Malaria Indicator Survey was provided by the Equatorial Guinea Ministry of Health and Social Welfare and the ethics committee of the London School of Hygiene and Tropical Medicine (approval number 5556). Written informed consent was sought from each participating adult and on behalf of participating children under 18 years of age.

### Sample collection

DBS samples were collected on Whatman filter papers (GE healthcare Ltd, Forest farm, Cardiff, UK; Product code: 11962089) from August to October 2019. On each filter paper are blood spots of 0.5 inches in diameter (n = 4), each spot representing a volume of approximately 50 μl of blood. Survey and laboratory data used in this study include: gender, rapid diagnostic test (RDT) diagnosis (*P. falciparum* or mixed infection), qPCR diagnosis (*P. falciparum* or mixed infection), travel history (previous two months), location (urban or rural, or Malabo, Baney, Luba, Riaba districts), age group in years (<5, 5–15, 15–18, > 18), and parasite density by qPCR (Low: Cq ≥ 20: High: Cq < 20). Household sampling was based on primary sampling units (PSUs) constructed from 1x1 km map-areas that make up the Bioko mapping grid [29]. PSUs were assigned to either a rural or an urban stratum based on the density of households within a community, with 25% and 5% of households sampled from each, respectively. All MIS participants (or their legal guardians, for participants <18 years of age) provided written informed consent. All identifiable data of MIS participants was excluded prior to distribution for analyses. Filter papers were selected for DNA extraction from 202 individuals with a positive RDT and reported fever and were shipped to the Malaria Research Program’s laboratory in February 2021.

Additionally, publicly available WGS data generated by the MalariaGEN *Plasmodium falciparum* Community Project [30] were downloaded from the Sequence Read Archive (SRA). Samples were selected from West, Central and East African countries as representative of their respective continental regions (S1 Table).

### DNA extraction

A DNA extraction method based on guanidine and silica purification protocols, and developed by the Malaria Research Program, at the University of Maryland Baltimore (UMB), was applied to selected DBS filter paper samples as described before [31]. Briefly, one circle/DBS was cutout, incubated, and submerged in lysis buffer for 2 hours at 65°C. Samples then underwent two washes before extracting DNA with TE buffer. Extracted DNA material was stored at -80°C until use.

### Polymerase chain reaction (PCR)

The Qiagen QuantiTect Multiplex PCR was used to conduct qPCR (Qiagen Sciences, Germantown, Maryland, USA). The master mix was prepared according to manufacturer’s instructions, but adapted to exclude ROX or UDG. RNAseq free water was used in all PCR reactions. The following PCR program was used: (1) 20 minutes at 50°C; (2) 15 minutes at 95°C; (3) 45 seconds at 94°C; (4) 75 seconds at 60°C. Steps 3 and 4 were repeated 45 times. For each sample, all PCR reactions were performed in duplicate. Samples were considered positive if the mean quantification cycles (Cq) of duplicate qPCR reactions was Cq < 40. If one sample result was reported as RDT positive and the corresponding qPCR result was negative, the qPCR assay was repeated to make a definitive conclusion, if necessary.

### Selective Whole Genome Amplification (sWGA)

Selective whole genome amplification (sWGA) was applied on extracted DNA with highest parasite density by Cq, as previously described [32,33]. In short, selected samples underwent a vacuum filtration step and were then amplified in 0.2 mL 96-well PCR plate with the following reaction mixture: 1X BSA, 1mM dNTPs, 2.5 µM of amplification primers, 1X Phi29 reaction buffer and 20 units of Phi29 polymerase. Primers are the same used in Oyola SO, et al. that preferentially bind at adequate distance and amplify *P. falciparum* genomes [33]. From each extraction, 17 µL of the DNA sample was added to the reaction mixture for a total of 50 µL final volume. Amplification occurred in a thermocycler with the following stepdown protocol: 35°C for 20 minutes, 34°C for 10 minutes, 33°C for 15 minutes, 32°C for 20 minutes, 31°C for 30 minutes, 30°C for 16 hours. The stepdown protocol was followed with a heating step at 65°C for 20 minutes and cooled to 4°C.

### Whole genome sequencing

Preparation of genomic DNA libraries was previously described [34]. To summarize, genomic DNA libraries were generated from amplified samples using the KAPA Library Preparation Kit (Kapa Biosystems, Woburn, MA), and then fragmented to approximately 200 base pair (bp) lengths. A modified version of the manufacturer’s protocol was used. AMPure XT beads were utilized to inform the library size selection. DNA concentration and fragment size was measured using the DNA High Sensitivity Assay on the LabChip GX (Perkin Elmer, Waltham, MA) tool. All sample libraries were uniquely barcoded, pooled and sequenced on 150 bp paired-end Illumina NovaSeq 6000 run (Illumina, San Diego, CA).

### Read mapping and identification of single nucleotide polymorphisms (SNPs)

Raw fastq files were mapped to the reference *P. falciparum* genome, 3D7, using bowtie2 [35]. BAM file processing followed GATK Best Practices [36,37]. Coverage and depth estimates from reads were generated using bedtools [38]. Variant calling of each sample was conducted utilizing the Haplotype Caller toolkit to generate genomic variant call format files (GVCF) and perform joint SNP (single nucleotide polymorphisms) calling. When appropriate, diploid calls were allowed since polyclonal infections were expected; otherwise major alleles were called (70% threshold) to genotype the most prevalent strain in the infection (polymorphic sites that did not reach the threshold were set to missing). Variant calls were filtered to omit potential false positive results with the following criteria: DP < 5, FS > 14.5, MQ < 20.0, QUAL<50. Additional filtering was performed to exclude rare allele events (frequency less than 0.05%), sites with >10% missingness, samples with >20% missing genotype values [39].

### Genetic diversity

Genetic variation in each geographic region was estimated by nucleotide diversity (*π*) (Vcftools v.0.1.16), the average pairwise difference between samples. Within-host diversity was measured by the F-statistic *F*_WS_ [40] using the R package moimix (v0.0.2.9001, https://bahlolab.github.io/moimix/), where diversity of *P. falciparum* in each sample was compared against the diversity of the entire sample set. Infections were considered polyclonal if *F*_WS_ < 0.95. *P*-values were calculated using Chi-squared test to determine statistical significance for differences in proportions of polyclonal infections, as determined by *F*_WS_, between African countries.

### Population structure

Principal components analysis (PCA) was applied to a data set of biallelic SNPs passing filtering criteria, to investigate the extent to which geographic origin contributes to differences between isolates at the genome-wide level. Clustering by PCA was estimated using the R package SNPRelate (v.1.28.0; https://github.com/zhengxwen/SNPRelate) [41,42]. Admixture analysis (ADMIXTURE v1.2) was used to obtain an estimate of contributions of inferred ancestral subpopulations to each sample. PCA and admixture data sets were pruned for sites in linkage disequilibrium (LD) (window size of 5 kbp, *r*^2^ ≥ 0.2) prior to analysis. Among samples with a clonal pair (i.e., nearly identical genomes), one sample was selected to represent the clonal group, and the other(s) excluded from the analysis. Chi-squared test was used to measure differences in composition among each inferred ancestral subpopulation between countries.

### Genetic differentiation

Wright’s fixation index, *F*_ST_, was applied to measure overall differentiation (mean *F*_ST_) between sampled parasite populations from different geographic regions, and to identify SNPs contributing to differences between populations [43]. Statistical significance of *F*_ST_ values were estimated empirically, with 5,000 permutations of samples by geographic region, using custom scripts (https://github.com/igs-jcsilva-lab) [44].

### Parasite relatedness

Overall relatedness between strains, as measured by identity-by-descent (IBD), was estimated using hmmIBD (version 2.0.0; https://github.com/glipsnort/hmmIBD) [45]. Prior to conducting IBD, samples were deconvoluted using dEploid v0.6-beta [46]. The strain accounting for the highest proportion of the sample was selected for downstream analysis. Previously published Python-based scripts were used to process files and run hmmIBD, and infomap (https://github.com/bguo068) [47], a community detection software, excluding IBD segments under 2cM and highly conserved IBD peaks to minimize bias [48]. Samples were considered related (siblings or clonal) if >25% of genomes were IBD [49]. Wilcoxon rank sum test was used to measure the difference in IBD distribution between sample sets.

### Visualization

The R package sf (license information: https://journal.r-project.org/archive/2018/RJ-2018-009/index.html) [50] was used to generate maps of Bioko that utilizes shapefiles from the geometrical operations (GEOS) (https://libgeos.org/) [51]. R (v4.1.3) was used with all appropriate R-based packages, such as ggplot2, to generate figures.

## Results

### Sample characteristics

**Fig 2:**
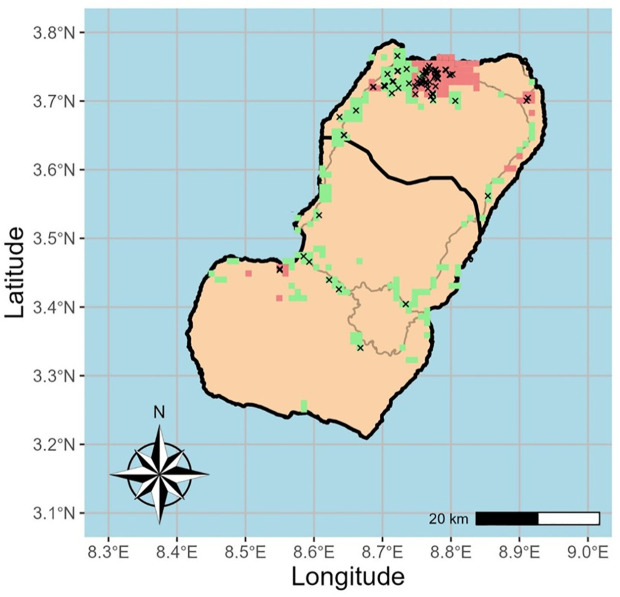
Urban and Rural Communities on Bioko Island. Map of Bioko Island with communities color-marked as urban (red) or rural (green). The urban/rural status was determined by the density of households within a community [14]. Black crosses represent geographical location of collection of all sequenced samples (n = 90). Shapefiles were obtained from the GEOs library (https://libgeos.org/).

Of the original 202 samples extracted, 160 were selected, prioritizing higher parasite density to improve WGS data quality and accounting for resource constraints. The DNA Cq of the 160 Bioko Island samples analyzed ranged between 24.3 and 38.9 (mean Cq = 33.7). Samples with highest parasite density were prioritized for sWGA (range: 24.3 – 38.2; mean Cq = 32.9). Whole genome shotgun sequencing (WGS) data was successfully generated for 90 samples. Of these, fifty-two samples were collected in urban communities compared to 38 from Bioko’s rural areas (Fig 2). An average of 28,276,493 total reads were generated per sample; on average, across samples, 75.4% of the reference 3D7 genome was genotyped with at least 5X read coverage (Figure A in S1 Data). Of the 90 samples, 16 samples were excluded due to inadequate quantity of mapped reads (< 8 million) and/or high missingness (>20% SNPs missing).

Bioko Island WGS data sets were combined with 992 sub-Saharan African WGS data sets after excluding samples based on genotype missingness (n = 25), and highly related, or clonal, clusters, where only one sample was included to represent the clonal group (n = 14). Public samples were selected from African countries representative of the continent’s West (Guinea, Mali, Burkina Faso, and Nigeria), Central region (Cameroon, Gabon and Democratic Republic of Congo) and East (Kenya, Tanzania, Malawi, and Mozambique). Variable sites in the highly polymorphic sub-telomeric genomic regions were excluded due to reduced accuracy [52,53]. After calling major alleles, a total of 1,076,963 biallelic SNPs were included, of which 375,921 (34.9%) were intergenic, 205,240 (19.1%) were synonymous and 426,551 (39.6%) were non-synonymous. Overall, average read coverage across all sites was 63.0X (59.9X for Bioko Island samples).

### Bioko island *P. falciparum* population harbors high levels of genetic variation

**Fig 3:**
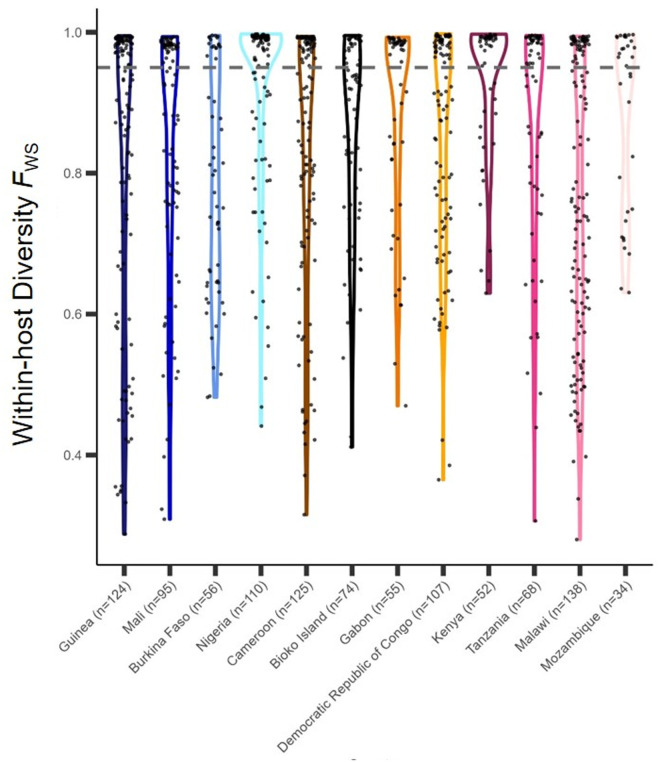
Within-host diversity as measured by F_WS_. Y-axis represents F_WS_ values of samples between 0 and 1. Isolates with F_WS_ value below 0.95 are inferred to be polyclonal (grey dotted line). Color groups denote the African region (West, Central, and East) as assigned by the WHO, and individual colors represent a country were samples were collected (x-axis).

Island settings with strong malaria elimination policies have seen a decrease in genetic diversity, and accompanying expansion of clonal parasites [6,54,55]. To describe the genetic variation of the *P. falciparum* population among Bioko Island samples (n = 74), nucleotide diversity and within-host diversity (*F*_WS_) were measured. Nucleotide diversity per site, estimated among variable sites only, was similar between Bioko district subgroups (*π*_Malabo city _= 0.0088 ± 0.04; *π*_Malabo suburbs _= 0.0086 ± 0.05; *π*_Baney_ = 0.0076 ± 0.06; *π*_Luba_ = 0.0079 ± 0.05). Nucleotide diversity was high among Bioko samples compared with that observed in nearby countries (all sites = 0.0087; nonsynonymous sites = 0.0088; synonymous sites = 0.0085) (Figure B in S1 Data). Within-host diversity measured by *F*_WS_ revealed the majority (46 out of 74) of isolates in Bioko Island were polyclonal (mean *F*_WS_ = 0.86) (Fig 3). While most polyclonal infections (n = 41) originated in Malabo communities, there was no significant difference in distribution of polyclonal infections between Malabo, Baney and Luba communities (*p*-value = 0.31). Further, the proportion of complex infections in Bioko Island did not significantly differ from the proportions seen in Cameroon (*p*-value = 1) or Gabon (*p*-value = 0.56), the closest African countries on the African mainland. When analyzing *F*_WS_ results by epidemiological subgroup, the odds of carrying a polyclonal infections was higher for children and travelers, consistent with previous epidemiological associations [11,14,21]. However, this trend was not statistically significant (Table A in S1 Data).

### Bioko Island *P. falciparum* population is not significantly differentiated from continental neighbors

**Fig 4:**
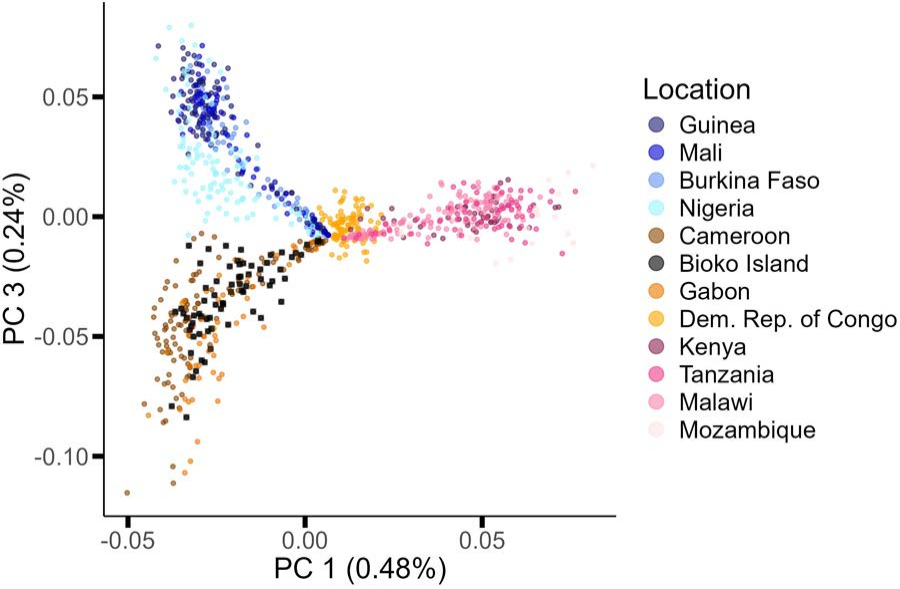
Principal Components Analysis (PCA) of Bioko and African *P. falciparum* strains. PC1 (x-axis) and PC3 (y-axis) illustrate the presence of four main clusters. PC1 separates East Africa from the DRC and both of those regions from countries in both West Africa and the Atlantic coast of Central Africa. In turn, PC3 separates West Africa from Central African populations from the Gulf of Guinea. The DRC sample set is centrally located between all clusters, but does not cluster with other Central African parasites. PC2 is not plotted since clustering pattern is not associated with geography but, instead, reflects the degree of genotype missingness. Color groups denote the African region (West, Central, and East) as assigned by WHO, and colors represent country were samples were collected.

To determine if Bioko’s island setting has led to an isolated and genetically distinct parasite population relative to its neighboring continental regions, PCA and admixture analysis were conducted. Variance within the sample set could be explained as geographical distance between country groups (West/Central *versus* East = 0.48%; West *versus* Central = 0.24%) (Fig 4). Bioko island parasites do not appear to form their own unique population relative to nearby continental parasite populations (*F*_ST Bioko – Cameroon_ = 0.01, p < 0.001; *F*_ST Bioko – Gabon_ = 0.01, p < 0.001), and little genetic differentiation was observed between Bioko Island parasites and other African parasite populations within the sample set (Table B in S1 Data). This suggests sufficient connectedness exists with Bioko Island that reduces any isolation effect of a geographical barrier.

**Fig 5:**
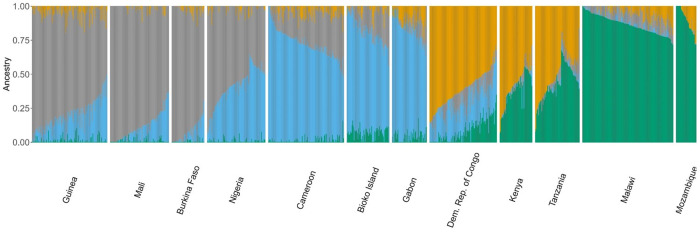
Admixture of Bioko and sub-Saharan parasites. Colors denote each ancestral subpopulation identified for K = 5, when cross validation error was lowest. Samples are represented as columns, Y-axis refers to the proportion of ancestry attributable to ancestral subpopulation, in each sample. Orange represents the subpopulation predominant in West Africa, green for Central Africa, grey for Democratic Republic of Congo and East Africa and blue for Southeast Africa. Yellow represents a subpopulation not predominant in any major African region.

The differentiation seen in the PCA analysis is corroborated by inferred ancestry. An admixture analysis was conducted, which resulted in the inference of the presence of five ancestral populations among the sub-Saharan sample set, with K = 5 corresponding to the lowest cross-validation error (Fig 5) (Figure C in S1 Data). Bioko Island parasites are characterized by a distinct ancestral population genomic signature that is common to Central-West African: most of the genome represents an ancestral population typical of the Gulf of Guinea (Fig 5, green population), followed by representation of ancestral populations more common in West Africa (in orange and yellow in Fig 5) and a small genomic proportion typical of Southeast Africa (Fig 5, blue and grey). When comparing the ancestral composition between Bioko Island, Cameroon and Gabon, geographical neighbors, the proportion of the blue subpopulation differed (Bioko Island – Cameroon – Gabon: p < 0.001). This subpopulation is predominant in Southeast Africa, and appears to contribute similar levels of ancestry to Bioko and Gabon populations, but not Cameroon (Bioko Island – Gabon: p = 0.43; Bioko Island – Cameroon: p < 0.001; Gabon – Cameroon: p = 0.004). Overall, Bioko parasites appear admixed, with very few (to no) genomes representing a single ancestral source.

### Greatest relatedness exists between Bioko and Gabonese parasite populations

**Fig 6:**
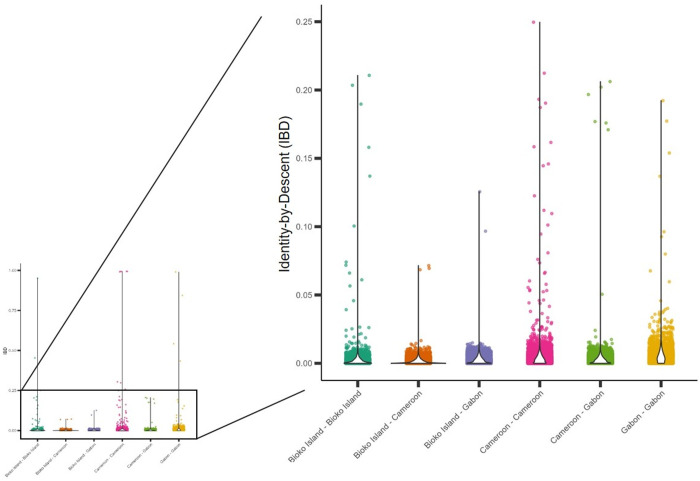
Identity-by-Descent (IBD) of *P. falciparum* pairwise comparisons between Bioko, Cameroon and Gabon. Violin plots illustrate the distribution of IBD values (y-axis) per comparison grouping (x-axis). Most pairwise comparisons are unrelated (IBD < 0.25) and illustrated in the right inset with magnification. All distributions are statistically differentiated from each other (Wilcox rank sum < 0.001).

To assess the impact of importation on the Bioko *P. falciparum* population, Cameroon and Gabon samples were used as a representative sample set for the suspected source of imported strains to Bioko due to their geographical proximity and admixture results. All samples were deconvoluted, and the predominant strain within each sample used. After merging and filtering, a total of 344,703 SNPs were used. Within Bioko, some strains were highly related (Figure D in S1 Data), but overall mean relatedness of Bioko Island strains was lower than seen within Cameroon or within Gabon (IBD_Bioko_ = 0.003; IBD_Cameroon_ = 0.005; IBD_Gabon_ = 0.006; p < 0.001) (Fig 6). Inter-group comparisons illustrated that relatedness of Bioko parasites was twice as high to Gabonese parasites as it was to those from Cameroon (IBD_Bioko-Gabon _= 0.002; IBD_Bioko-Cameroon _= 0.001; p < 0.001), and approximately 40% of Bioko parasites formed subpopulations with Gabonese parasites. A similar observation was made when conducting PCA with the same sample set, where Bioko Island strains appear to cluster more closely with Gabon than with Cameroon (Figure E in S1 Data). Ultimately, the *P. falciparum* population within Bioko Island is genetically diverse and composed in largely unrelated parasites, which have marginally higher relatedness to the Gabonese than to the Cameroonian *P. falciparum* population.

### Malabo city parasites differ significantly from those in Baney and Luba in loci associated with cellular adhesion, invasion and sequestration

The human demography of Bioko Island, where the majority of residents live in Malabo city and the surrounding communities are mostly rural, creates the opportunity for a structured *P. falciparum* population on the island. Previous MIS results reported differential measurements of malaria prevalence between urban and rural communities, with higher prevalence in rural communities [10,21]. To determine if multiple parasite populations existed on Bioko Island, we investigated possible genetic differentiation between Bioko-specific geographical subgroups using *F*_ST_. When stratifying samples according to district (Malabo, Baney and Luba), there was some suggestion of overall genetic differentiation between districts, however results were not statistically supported (*F*_ST Malabo vs Baney _= 0.04, p-value = 0.73; *F*_ST Malabo city vs Luba _= 0.04, p-value = 0.63). To identify genomic loci contributing to differentiation within Bioko populations, *F*_ST_ per site was calculated between Malabo city, Baney and Luba. Among a total of 163,291 SNPs (Figure F in S1 Data), Baney and Luba consistently differed after 5,000 permutations (p < 0.05) from Malabo city isolates at loci associated with red blood cell invasion (*clag3.2* - PF3D7_0302200; *msp* - PF3D7_1035600), hepatocyte invasion (DBL-containing protein - PF3D7_0113800), and parasite sequestration (*var2csa* - PF3D7_1200600).

## Discussion

Considering its central location between Nigeria and the Democratic Republic of Congo (DRC), the two countries globally suffering the highest number of malaria cases in 2022 (27% and 12%, respectively), Equatorial Guinea, including Bioko island, represents an important reference point regarding the disease and potential effect of control interventions within Central Africa [1]. As sequencing becomes more accessible, national malaria control programs are incorporating sequencing methodologies as an essential molecular tool to provide improved genotyping accuracy and robustness to supplement traditional epidemiological approaches to inform control and elimination strategies [24,56–58]. As part of the BIMEP’s effort to eliminate malaria from the island of Bioko, WGS data was generated from the 2019 MIS field samples to describe the parasite population at the genomic level.

*P. falciparum* on Bioko Island has genomic epidemiological features characteristic of endemic areas with moderate transmission [1]. For example, most infections were polyclonal; high rates of polyclonality is often a proxy measurement of high transmission intensity since it reflects high rates of biting by infected mosquitoes [59,60]. Therefore, and despite large investments in control interventions that resulted in a significant decrease in malaria incidence, complexity of infection on Bioko Island remains similar to other regions in Africa with higher malaria incidence [6,61,62], suggesting that sustained importation contributes to genetic diversity on Bioko [63,64]. This is consistent with the high genetic diversity of the *P. falciparum* population in Bioko Island and its significant relatedness to others in the African mainland, and human migration is likely a significant contributor to on-island transmission and local genetic diversity of *P. falciparum*. Nevertheless, the observation of many polyclonal infections suggest other factors such as high mosquito biting rates and breeding sites continue to sustain local transmission [65,66].

The presence, in the genome composition of Bioko samples – as well as in those from Gabon and DRC, of a contribution from an inferred ancestral subpopulation predominant in Southeast Africa hints at a closer connection between those regions that does not extend to Cameroon. This is further supported by the IBD analysis, where Bioko parasites had higher relatedness with Gabonese parasites than with Cameroon. Interestingly, the Gabonese samples used were collected in Wouleu-Ntem province, which has an extensive border with Equatorial Guinea, suggesting this Gabonese sample set may also be representative of Eastern Rio Muni, a frequent travel destination of off-island travelers [14]. There are close cultural and ethnic ties between Equatorial Guinea and Gabon, as the predominant ethnic group in both countries is the Fang tribe [67–69]. Although speculative, this cultural connection may result in increased border crossings and trade in Eastern Rio Muni that then contributes to Bioko’s parasite population when infected travelers return to the island.

The goal to eliminate malaria from Bioko Island rested in part on the assumption that its island status conferred a significant level of protection from importation from surrounding regions. However, BIMEP’s malaria control efforts are still hampered by significant importation. Similar to Bioko, the island of Zanzibar, in Tanzania, and the island country of São Tomé and Principe, have been unable to eliminate malaria, following steep reductions in malaria incidence since the turn of the century [1]. In both cases, malaria importation from higher transmission areas is a key obstacle to elimination [70–73]. However, unlike these other cases, the rate of malaria incidence in Rio Muni, the most likely source of imported strains to Bioko [14,15], is substantively higher than that in most of the expected sources of importation to Zanzibar and São Tomé and Principe [74–76]. Further, the lack of malaria control in Rio Muni since 2011 likely means that transmission is high throughout the entire region [17]; consequently, any traveler to the mainland may be exposed. While the challenges faced by the BIMEP in terms of case importation are not unique, the proximity and exposure to regions with high transmission make them especially acute. Results from Bioko showcase the significance of importation to the island from an area with no malaria control presence, likely the largest hindrance to progress. Reversal of the current situation might require Equatorial Guinea’s significant investment in malaria control to extend to all regions of the country and, in a broader sense, to collaboration with governments in other malaria endemics regions surrounding its borders.

This study had some limitations. No DBS samples have been collected from the continental region of Equatorial Guinea, the most likely source of importation to Bioko, and which may help better distinguish between local and imported strains. Unfortunately, this was unavoidable as, to our knowledge, no MIS has occurred in the Rio Muni since 2011. Also, parasite WGS data was only generated from individuals with positive RDT and fever, to optimize the chances of obtaining good quality sequencing data; however, this bias may have resulted in an over-sampling of complex infections. Sample size was also limited due to resource constraints and could have affected results, but impact is thought to be minimal since whole genome data provide thousands of data points for analysis. Ultimately, this study follows accepted methodologies [6,61,63,77], and accounts for the limitations within its analysis and interpretations.

Using sWGA, high-quality WGS *P. falciparum* data was generated from DBS collected during the 2019 MIS on Bioko Island, Equatorial Guinea, to describe local transmission dynamics and estimate the extent of differentiation between the local parasite population and those from nearby continental regions. Locally, there did not appear to be population structure. We found no significant genome-wide genetic differentiation between the Bioko Island parasite population and those neighboring countries in mainland Central-West Africa region. Most notably, the flow of parasites appeared marginally higher between Bioko and Gabonese populations, compared to Cameroon, possibly reflecting more frequent human mobility between Bioko and the Eastern Rio Muni. Considerable reduction in island-wide malaria transmission is needed, and importation to the island needs to be addressed to ensure continued progress. Additional sequencing data from parasites on Bioko and the Equatorial Guinea mainland, ideally from both symptomatic and asymptomatic infections, might provide further insights into local transmission dynamics and temporal changes. The current WGS data set provides an informative snapshot of regional relationships among *P. falciparum* populations, successfully sequencing parasites from field samples, and illustrated the effect imported strains may be having on local malaria elimination efforts. Ultimately, for there to be sustained, prolonged progress towards malaria elimination, the BIMEP could consider targeted screening and control interventions for travelers to the island, and if possible, reestablish malaria control in Rio Muni.

## Supporting information

S1 Data **Figure A:** Cq value of sequenced samples from Bioko Island 2019 MIS as measured by Multiplex PCR (Qiagen Sciences, Germantown, MD, USA) prior to selective whole genome amplification and total reads mapping to P. falciparum reference genome (Pf3D7). Multiplex PCR also amplifies non-falciparum Plasmodium species and is denoted by the color of each point (i.e., Infection type) where Pf = P. falciparum, Pm = P. malariae, and Po = P. ovale to identify co-infection samples. The size of the each point reflects the proportion of the genome with at least 5X coverage from the sequenced reads. Infections with a Cq value below 32 (i.e., higher parasite density) typically resulted a higher proportion of reads mapping to the P. falciparum reference gnome and optimal coverage. Sixteen Bioko samples were dropped due to a lack of mapped reads to P. falciparum or insufficient genome coverage. **Figure B:** Mean nucleotide diversity (π) per-site in genome-wide variable sites was estimated for all sites (ALL), nonsynonymous sites (NONSYN), and synonymous sites (SYN) among a sample set of sub-Saharan African P. falciparum strains and the reported country-wide P. falciparum prevalence due the year of sample collection. Prevalence was determined from reported prevalence estimates in the 2023 WHO World Malaria Report. Black triangle denotes measurements of Bioko parasites. **Figure C:** Plot of CV error from admixture analysis estimations for each population (K) among sample set of sub-Saharan African and Bioko Island samples. The results from the admixture model with lowest CV error was selected (K = 5). **Figure D:** Map of Bioko Island with communities marked as urban (red) rural (green) assignments. Crosses mark the geographical location of each sequenced sample from the 2019 MIS. Lines drawn between sample pairs represent high relatedness, as measured by IBD (IBD > 0.25). Shapefiles were obtained from the GEOs library (https://libgeos.org/). **Figure E:** Principal Components Analysis (PCA) of Bioko Island (Black), Cameroon (pink) and Gabon (blue) P. falciparum strains. PC1 (x-axis) and PC2 (y-axis) illustrate how Bioko Island strains appear to cluster more closely with Gabonese strains, suggestive of a closer genetic link than with Cameroonian strains. All samples were deconvoluted and the predominant strains used. The sample set used to generate the PCA included 344,703 SNPs. **Figure F:** Differentiation between P. falciparum subpopulations on Bioko Island. Allele frequency distribution was measured between three Bioko subpopulations based on location: A. Malabo vs Baney (Total SNPs = 1,699); B. Malabo vs Luba (Total SNPs = 1,420); C. Baney vs Luba (Total SNPs = 790) by the fixation index (F_ST_). A total of 163,291 SNPs were included in each sample set with heterozygous sites included and minor allele excluded. Dotted line in each panel represents the 10% of highest F_ST_ values per sample set. **Table A:** Multi-variate logistic regression analysis of epidemiological subgroups associated with polyclonal infections (as determined by the F_ws_ statistic) as a proxy of transmission intensity. χ^2^ test was used to determine p-values. R v4.1.3 was used with base package stats to generate logistic model. Subgroups associated with higher transmission intensity were individuals under 18 years old, travelers, and mixed (non-falciparum) infections. Although no significant results were observed, trends agree with previous epidemiological models on Bioko Island [11,14,21]. **Table B:** Estimated F_ST_ between all African countries among the sampled P. falciparum populations. Values were generated using vcftools (v.0.1.16) (4). Bioko Island associated F_ST_ values are highlighted in yellow. From these values, little differentiation is observed between Bioko Island and other African parasite populations, and suggests Bioko Island parasites do not form a unique population within the African context.
(DOCX)

S2 Data De-identified survey data collected during the 2019 MIS conducted on Bioko Island, Equatorial Guinea. Data is linked with the corresponding WGS data.
(XLSX)

S1 Table List of WGS data with BioSample ID. WGS data was generated by the MalariaGEN Plasmodium falciparum Community Project [30] and downloaded from the Sequence Read Archive (SRA).
(XLSX)

S1 Checklist Inclusivity in global research. (DOCX)
