# Supplementary material for: Regular Plasmodium falciparum importation onto Bioko Island, Equatorial Guinea, hampers malaria elimination from the island
Source: PLOS Glob Public Health. 2025 Aug 19;5(8):e0004999. doi: 10.1371/journal.pgph.0004999 (PMC12364312; doi:10.1371/journal.pgph.0004999)
Supplement: S1 Data — Figure A: Cq value of sequenced samples from Bioko Island 2019 MIS as measured by Multiplex PCR (Qiagen Sciences, Germantown, MD, USA) prior to selective whole genome amplification and total reads mapping to P. falciparum reference genome (Pf3D7). Multiplex PCR also amplifies non-falciparum Plasmodium species and is denoted by the color of each point (i.e., Infection type) where Pf = P. falciparum, Pm = P. malariae, and Po = P. ovale to identify co-infection samples. The size of the each point reflects the proportion of the genome with at least 5X coverage from the sequenced reads. Infections with a Cq value below 32 (i.e., higher parasite density) typically resulted a higher proportion of reads mapping to the P. falciparum reference gnome and optimal coverage. Sixteen Bioko samples were dropped due to a lack of mapped reads to P. falciparum or insufficient genome coverage. Figure B: Mean nucleotide diversity (π) per-site in genome-wide variable sites was estimated for all sites (ALL), nonsynonymous sites (NONSYN), and synonymous sites (SYN) among a sample set of sub-Saharan African P. falciparum strains and the reported country-wide P. falciparum prevalence due the year of sample collection. Prevalence was determined from reported prevalence estimates in the 2023 WHO World Malaria Report. Black triangle denotes measurements of Bioko parasites. Figure C: Plot of CV error from admixture analysis estimations for each population (K) among sample set of sub-Saharan African and Bioko Island samples. The results from the admixture model with lowest CV error was selected (K = 5). Figure D: Map of Bioko Island with communities marked as urban (red) rural (green) assignments. Crosses mark the geographical location of each sequenced sample from the 2019 MIS. Lines drawn between sample pairs represent high relatedness, as measured by IBD (IBD > 0.25). Shapefiles were obtained from the GEOs library (https://libgeos.org/). Figure E: Principal Components Analysis (PCA) of Bioko I [file pgph.0004999.s001.docx]

**Regular *Plasmodium falciparum* importation onto Bioko Island, Equatorial Guinea, hampers malaria elimination from the island.**

Thomas C. Stabler*^1, 2^, Ankit Dwivedi^3^, Bing Guo^4^, Biraj Shrestha^4^, Sudhaunshu Joshi^4^, Matilde Riloha Rivas^5^, Olivier Tresor Donfack^6^, Carlos A. Guerra^7^, Guillermo A. García^7^, Claudia Daubenberger^1, 2^, Joana C. Silva*^3, 8 , 9^

^1^Department of Medical Parasitology and Infection Biology, Swiss Tropical and Public Health Institute, Basel, Switzerland

^2^University of Basel, Basel, Switzerland

^3^Institute for Genome Sciences, University of Maryland School of Medicine, Baltimore, Maryland, USA

^4^Malaria Research Program, Center for Vaccine Development and Global Health, University of Maryland Baltimore, Baltimore, MD, USA

^5^Equatorial Guinea Ministry of Health and Social Welfare, Malabo, Equatorial Guinea

^6^Medical Care Development Global Health, Malabo, Equatorial Guinea

^7^Medical Care Development Global Health, Silver Spring, Maryland, USA

^8^Department of Microbiology and Immunology, University of Maryland School of Medicine, Baltimore, Maryland, USA

^9^Global Health and Tropical Medicine (GHTM), Instituto de Higiene e Medicina Tropical (IHMT), Universidade NOVA de Lisboa (NOVA), Lisbon, Portugal

**Supplementary figures/tables**


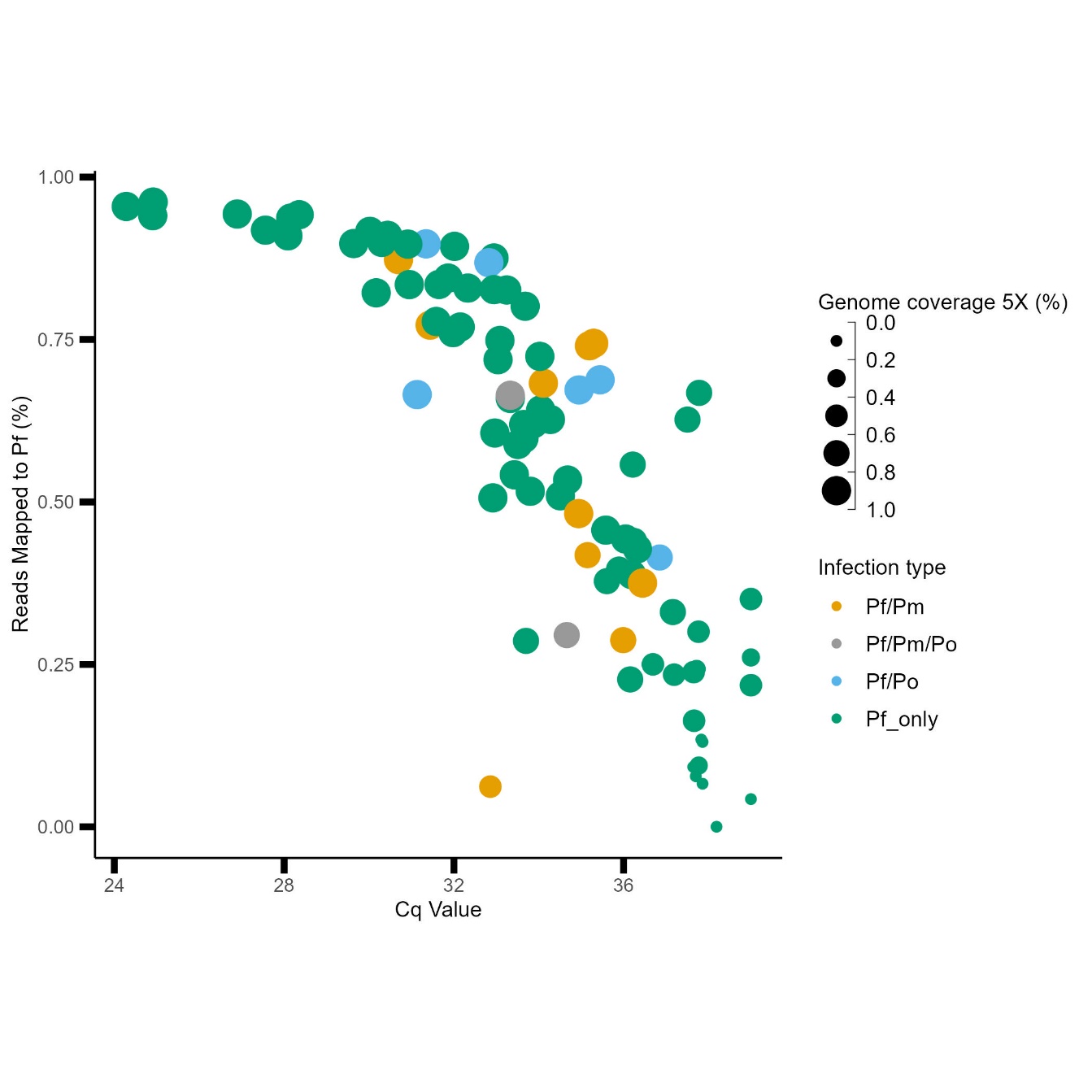


Supplementary Figure S1. Cq value of sequenced samples from Bioko Island 2019 MIS as measured by Multiplex PCR (Qiagen Sciences, Germantown, MD, USA) prior to selective whole genome amplification and total reads mapping to P. falciparum reference genome (Pf3D7). Multiplex PCR also amplifies non-falciparum Plasmodium species and is denoted by the color of each point (i.e. Infection type) where Pf = P. falciparum, Pm = P. malariae, and Po = P. ovale to identify co-infection samples. The size of the each point reflects the proportion of the genome with at least 5X coverage from the sequenced reads. Infections with a Cq value below 32 (i.e. higher parasite density) typically resulted a higher proportion of reads mapping to the P. falciparum reference gnome and optimal coverage. Sixteen Bioko samples were dropped due to a lack of mapped reads to P. falciparum or insufficient genome coverage.


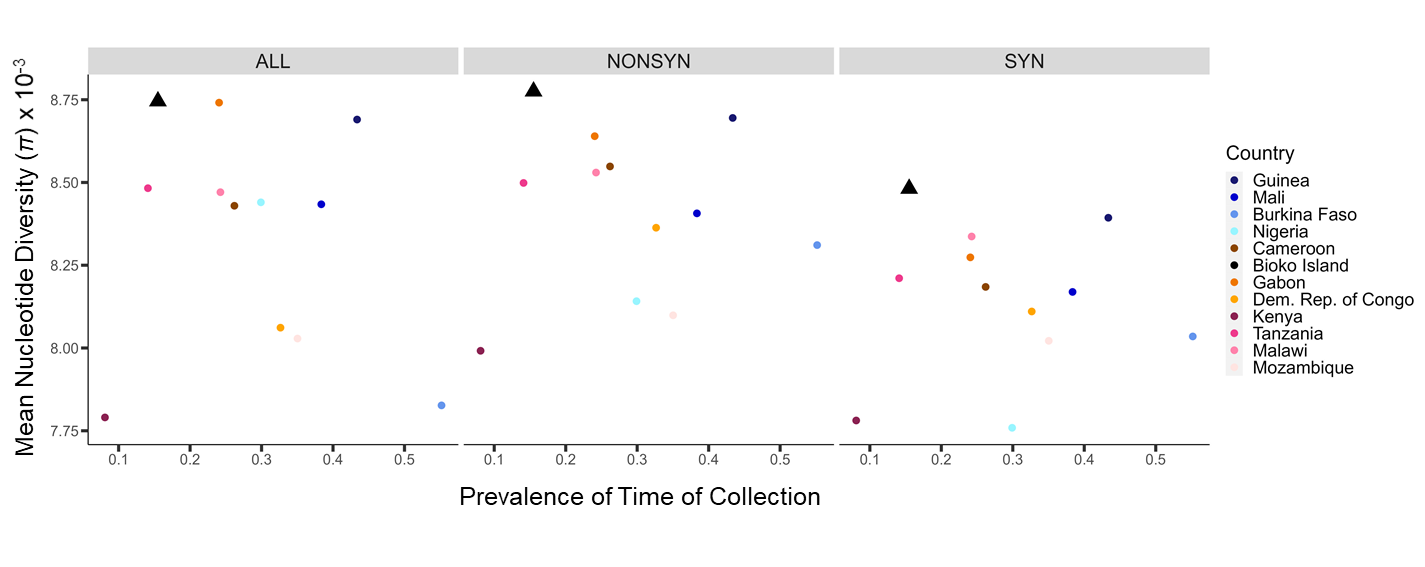


Supplementary Figure S2. Mean nucleotide diversity (π) per-site in genome-wide variable sites was estimated for all sites (ALL), nonsynonymous sites (NONSYN), and synonymous sites (SYN) among a sample set of sub-Saharan African P. falciparum strains and the reported country-wide P. falciparum prevalence due the year of sample collection. Prevalence was determined from reported prevalence estimates in the 2023 WHO World Malaria Report. Black triangle denotes measurements of Bioko parasites.


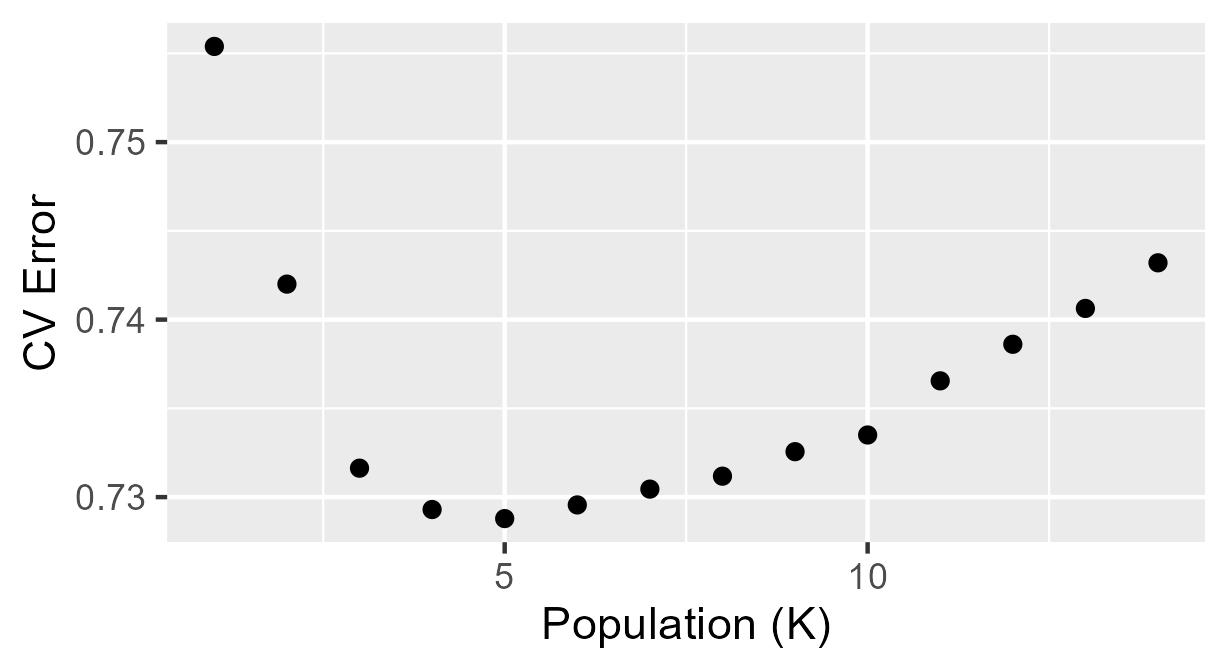


Supplementary Figure S3. Plot of CV error from admixture analysis estimations for each population (K) among sample set of sub-Saharan African and Bioko Island samples. The results from the admixture model with lowest CV error was selected (K=5).


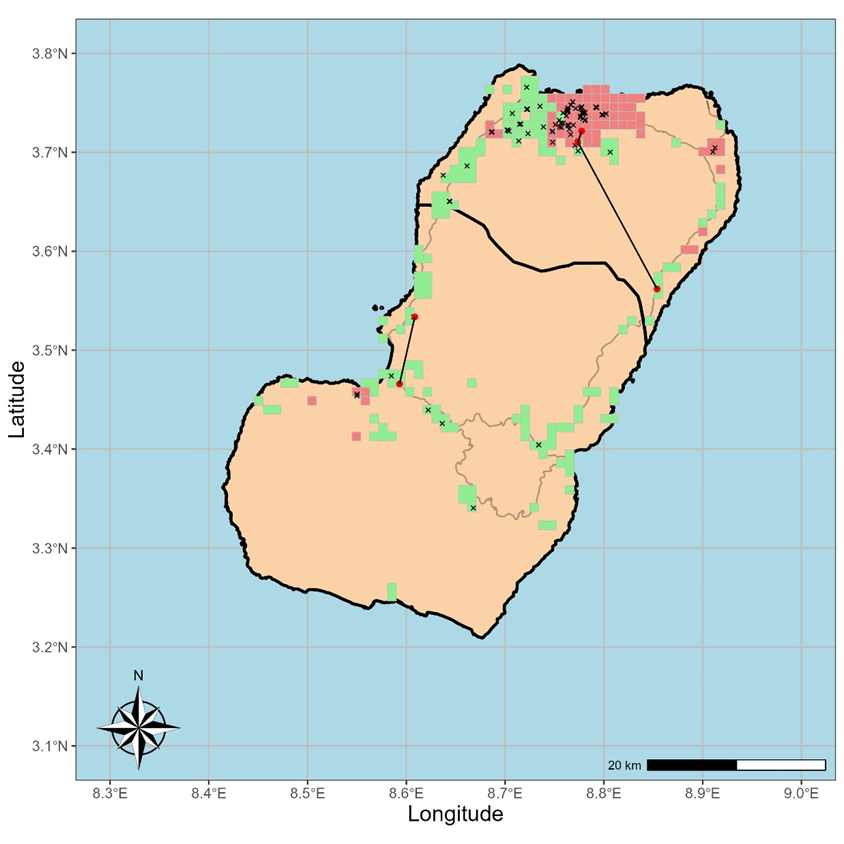


Supplementary Figure S4. Map of Bioko Island with communities marked as urban (red) rural (green) assignments. Crosses mark the geographical location of each sequenced sample from the 2019 MIS. Lines drawn between sample pairs represent high relatedness, as measured by IBD (IBD > 0.25). Shapefiles were obtained from the GEOs library (<https://libgeos.org/>).


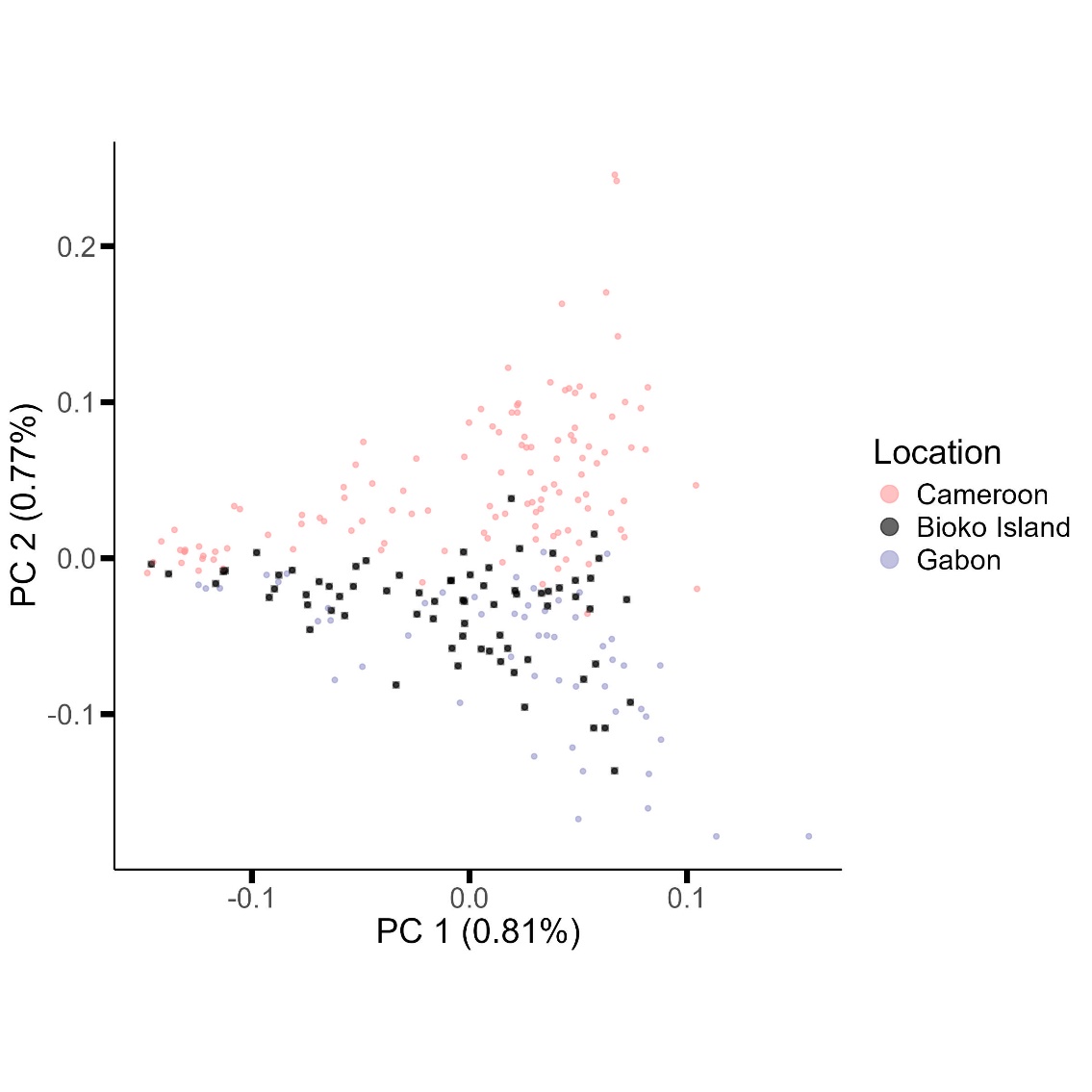


Supplementary Figure S5. Principal Components Analysis (PCA) of Bioko Island (Black), Cameroon (pink) and Gabon (blue) P. falciparum strains. PC1 (x-axis) and PC2 (y-axis) illustrate how Bioko Island strains appear to cluster more closely with Gabonese strains, suggestive of a closer genetic link than with Cameroonian strains. All samples were deconvoluted and the predominant strains used. The sample set used to generate the PCA included 344,703 SNPs.


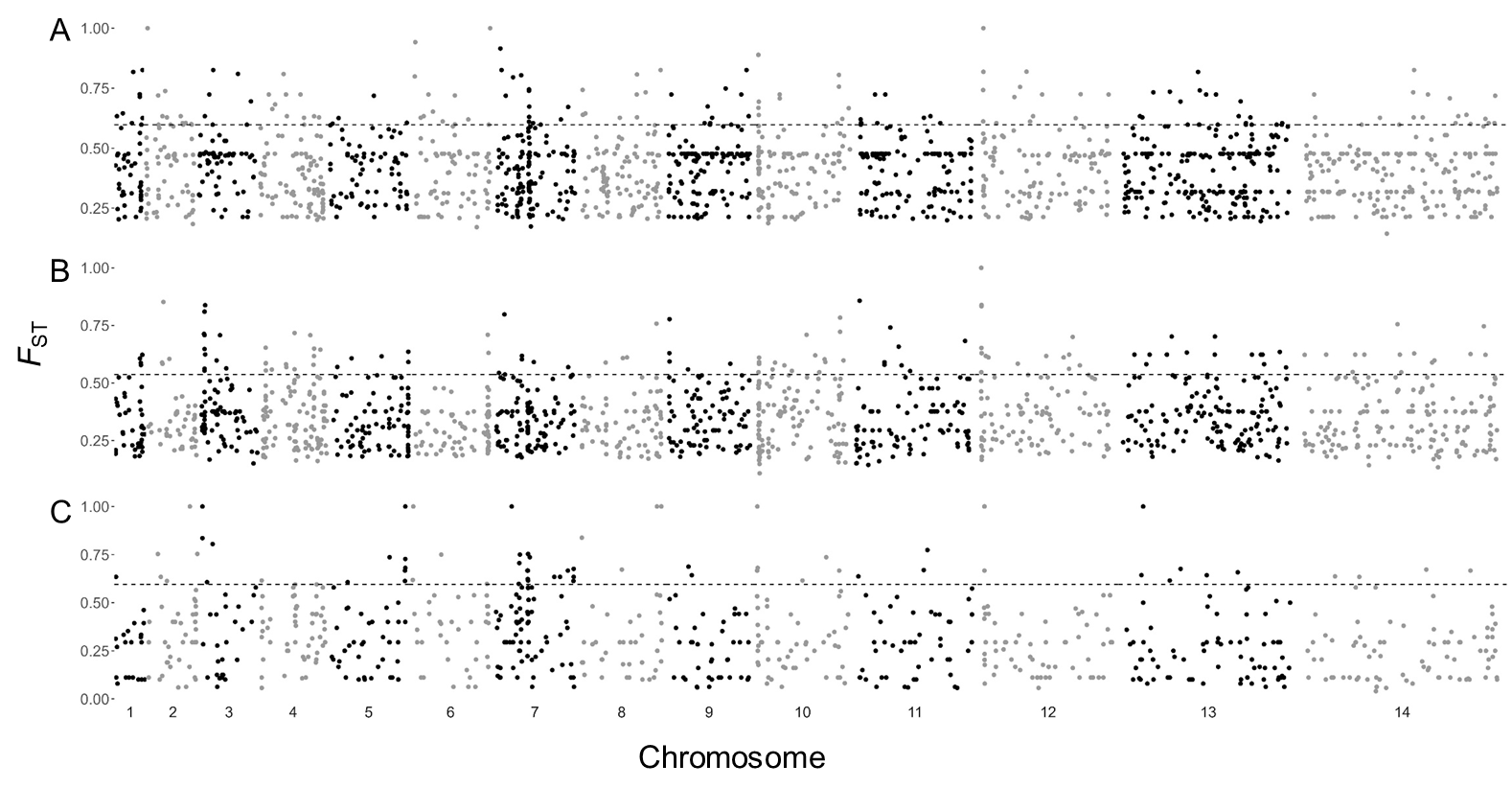


Supplementary Figure S6. Differentiation between P. falciparum subpopulations on Bioko Island. Allele frequency distribution was measured between three Bioko subpopulations based on location: A. Malabo vs Baney (Total SNPs = 1,699); B. Malabo vs Luba (Total SNPs = 1,420); C. Baney vs Luba (Total SNPs = 790) by the fixation index (F_ST_). A total of 163,291 SNPs were included in each sample set with heterozygous sites included and minor allele excluded. Dotted line in each panel represents the 10% of highest F_ST_ values per sample set. Supplementary Table S1. Multi-variate logistic regression analysis of epidemiological subgroups associated with polyclonal infections (as determined by the F_ws_ statistic) as a proxy of transmission intensity. χ^2^ test was used to determine p-values. R v4.1.3 was used with base package stats to generate logistic model. Subgroups associated with higher transmission intensity were individuals under 18 years old, travelers, and mixed (non-falciparum) infections. Although no significant results were observed, trends agree with previous epidemiological models on Bioko Island (1-3).

| **Association to Polyclonal infections** | | | | |
| --- | --- | --- | --- | --- |
| **Variable** | **Subgroup** | **N (% polyclonal)** | **OR (95% CI)** | ***p*-value** |
| Gender | Male | 38 (63.2) | 1 (base) | 0.67 |
|  | Female | 36 (55.6) | 0.69 (0.25, 1.86) |  |
| RDT | Pf | 21 (52.4) | 1 (base) | 0.60 |
|  | Mixed | 53 (62.3) | 1.33 (0.43, 4.06) |  |
| PCR | Pf | 58 (55.2) | 1 (base) | 0.25 |
|  | Mixed | 16 (75.0) | 2.74 (0.79, 11.65) |  |
| Travel | Non-traveler | 50 (56.0) | 1 (base) | 0.53 |
|  | Traveler | 24 (66.7) | 1.98 (0.63, 6.80) |  |
| Location | Urban | 41 (63.4) | 1 (base) | 0.59 |
|  | Rural | 33 (54.5) | 0.70 (0.24, 1.99) |  |
| Age | Adult | 34 (52.9) | 1 (base) | 0.41 |
|  | Under 18 | 40 (65.0) | 2.13 (0.75, 6.35) |  |
| Parasitemia | Low | 43 (62.8) | 1 (base) | 0.65 |
|  | High | 31 (54.8) | 0.66 (0.24, 1.84) |  |
|  |  |  |  |  |

Supplementary Table S2. Estimated F_ST_ between all African countries among the sampled P. falciparum populations. Values were generated using vcftools (v.0.1.16) (4). Bioko Island associated F_ST_ values are highlighted in yellow. From these values, little differentiation is observed between Bioko Island and other African parasite populations, and suggests Bioko Island parasites do not form a unique population within the African context.

**References**

1. García GA, Janko M, Hergott DEB, Donfack OT, Smith JM, Mba Eyono JN, et al. Identifying individual, household and environmental risk factors for malaria infection on Bioko Island to inform interventions. Malaria Journal. 2023;22(1):72.

2. Guerra CA, Kang SY, Citron DT, Hergott DEB, Perry M, Smith J, et al. Human mobility patterns and malaria importation on Bioko Island. Nature Communications. 2019;10(1):2332.

3. Guerra CA, Citron DT, García GA, Smith DL. Characterising malaria connectivity using malaria indicator survey data. Malaria Journal. 2019;18(1):440.

4. Weir BS, Cockerham CC. Estimating F-Statistics for the Analysis of Population Structure. Evolution. 1984;38(6):1358-70.
